# *Citrus aurantium* L. essential oil exhibits anxiolytic-like activity mediated by 5-HT_1A_-receptors and reduces cholesterol after repeated oral treatment

**DOI:** 10.1186/1472-6882-13-42

**Published:** 2013-02-23

**Authors:** Celso A R A Costa, Thaís C Cury, Bruna O Cassettari, Regina K Takahira, Jorge C Flório, Mirtes Costa

**Affiliations:** 1 Department of Pharmacology, Institute of Biosciences, Unesp - Univ Estadual Paulista, P.O. Box 510, 18618-970, Botucatu, São Paulo, Brazil; 2 Department of Pathology, School of Veterinary Medicine and Animal Science, Unesp - Univ Estadual Paulista, Laboratório Clínico Veterinário, 18618-970, Botucatu, SP, Brazil; 3 Department of Pathology, School of Veterinary Medicine, USP - University of São Paulo, Av. Orlando Marques de Paiva, 87, São Paulo, SP, Brazil

**Keywords:** Anxiolytic, *Citrus aurantium*, Essential oil, Mice, Neurochemistry, Serotonin

## Abstract

**Background:**

The current treatments for anxiety disorders and depression have multiple adverse effects in addition to a delayed onset of action, which has prompted efforts to find new substances with potential activity in these disorders. *Citrus aurantium* was chosen based on ethnopharmacological data because traditional medicine refers to the *Citrus* genus as useful in diminishing the symptoms of anxiety or insomnia, and *C*. *aurantium* has more recently been proposed as an adjuvant for antidepressants. In the present work, we investigated the biological activity underlying the anxiolytic and antidepressant effects of *C*. *aurantium* essential oil (EO), the putative mechanism of the anxiolytic-like effect, and the neurochemical changes in specific brain structures of mice after acute treatment. We also monitored the mice for possible signs of toxicity after a 14-day treatment.

**Methods:**

The anxiolytic-like activity of the EO was investigated in a light/dark box, and the antidepressant activity was investigated in a forced swim test. Flumazenil, a competitive antagonist of benzodiazepine binding, and the selective 5-HT_1A_ receptor antagonist WAY100635 were used in the experimental procedures to determine the mechanism of action of the EO. To exclude false positive results due to motor impairment, the mice were submitted to the rotarod test.

**Results:**

The data suggest that the anxiolytic-like activity observed in the light/dark box procedure after acute (5 mg/kg) or 14-day repeated (1 mg/kg/day) dosing was mediated by the serotonergic system (5-HT_1A_ receptors). Acute treatment with the EO showed no activity in the forced swim test, which is sensitive to antidepressants. A neurochemical evaluation showed no alterations in neurotransmitter levels in the cortex, the striatum, the pons, and the hypothalamus. Furthermore, no locomotor impairment or signs of toxicity or biochemical changes, except a reduction in cholesterol levels, were observed after treatment with the EO.

**Conclusion:**

This work contributes to a better understanding of the biological activity of *C*. *aurantium* EO by characterizing the mechanism of action underlying its anxiolytic-like activity.

## Background

Since the 1960s, the benzodiazepines, which improve GABAergic neurotransmission, have been used extensively to treat anxiety disorders. These compounds have shown a clear efficacy, leading to their becoming the most widely prescribed drugs worldwide [1]. However, many patients fail to adequately respond to treatment [2], and the side effects of benzodiazepines include sedation, muscle relaxation, potentiation of the depressant effects of ethanol, anterograde amnesia, and addiction [3]. Buspirone, a non-benzodiazepine azapirone agent that was introduced in the late 1980s as a novel therapeutic agent for the treatment of anxiety, functions by interacting with the 5-hydroxytryptamine_1A_ (5-HT_1A_) receptor subtype [4]. However, the delayed onset of efficacy of buspirone (1–4 weeks), in contrast to the rapid effects observed with benzodiazepines [5], has encouraged researchers to search for a robust anxiolytic compound that has fewer side effects than benzodiazepines and a more immediate onset of action than azapirones.

On the other hand, the various therapeutic pharmaceutical classes (monoamine oxidase inhibitors, tricyclics and selective serotonin reuptake inhibitors) used to treat depression depend on monoamine (noradrenaline, dopamine and mainly serotonin) availability in the brain. Despite the large variety of medicines available, roughly half of the patients seeking treatment do not respond to classical antidepressants, and at least 2 weeks are necessary for symptoms to be substantially ameliorated [6,7]. Antidepressants cause a robust set of side effects, such as weight gain, constipation, memory disorders, and sleep and sexual disorders, which vary in frequency and intensity among the different classes of antidepressants [8].

Thus, the presence of multiple adverse side effects that affect a considerable proportion of patients, as well as the delayed onset of effects, drive the efforts to identify new substances that can potentially treat anxiety and depression [9,10].

Traditionally, populations in several countries have relied on preparations obtained from *Citrus* species to treat problems related to the nervous system, especially symptoms of anxiety or insomnia [11-15]. Sedative and anxiolytic-like effects have been described for the essential oil (EO) obtained from the peel of *Citrus aurantium* L. [16,17]. In humans, drops of *C*. *sinensis* EO scattered in the lobby of a dental office reduced the anxiety levels of patients, specifically women [18]. Administered orally, the EO derived from the petals and stamens of *C*. *aurantium* reduced the preoperative anxiety of patients scheduled for elective minor surgery [19].

More recently, preparations from *Citrus* species have also been investigated for antidepressant activity in both rodents and humans. EO preparations made from the leaves of *C*. *maxima*[20] and *C*. *limon*[21] decreased the immobility time of mice in the forced swim and tail suspension tests, strengthening the suggestion that citrus fragrance can reduce the dose of antidepressants required to treat depressive subjects [22].

Our group found similar effects in experiments investigating the oral treatment of mice with the EOs obtained from the peels of *C*. *aurantium*[23,24], *C*. *latifolia* or *C*. *reticulate*[25]. The EOs increased the amount of time spent in the open arms of the elevated plus maze [23] and the lighted compartment in the light/dark box test, in addition to reducing the number of hidden marbles in the marble burying test [24,25]. While the elevated plus maze and the light/dark box are sensitive to drugs such as benzodiazepines and are models for generalized anxiety disorder [26], the marble burying test is a model for obsessive-compulsive disorder [27], which benefits from treatment with antidepressants. Thus, the activity profile observed for EOs from *Citrus* species denotes a wide spectrum of action given that the EOs were active in experimental models sensitive to both anxiolytic and antidepressant drugs.

Considering this background, the aim of the present work was to investigate the putative mechanism of the anxiolytic-like effect and identify any neurochemical changes in specific cerebral areas that result from acute treatment with *C*. *aurantium* EO. To better characterize their activity, the EO was also evaluated in anxiety tests after a repeated 14-day oral treatment, where changes in body weight, the integrity of the locomotor system and serum biochemical parameters were monitored. Finally, the EO was evaluated in experimental procedures related to depressive disorders after oral or inhaled treatment.

## Methods

### Plant materials

*C*. *aurantium* (Rutaceae) ripe fruits were harvested between April and June of 2009 from adult plants in an orchard at the Department of Botany, Institute of Biosciences, UNESP, Botucatu. The plant was identified in the BOTU Herbarium of the Department of Botany, UNESP, where a voucher specimen (#23123) had been deposited.

### EO extraction and phytochemical analysis

Immediately after harvesting the fruits were peeled and the fresh peels were processed with a Clevenger apparatus, and the EO was obtained through hydrodistillation while protected from light and heat. The EO was then stored until use in the behavioral assays. Afterwards, an aliquot was separated, and the EO was analyzed by gas chromatography coupled with mass spectrometry as previously described [24].

### Animals

All of the experiments were conducted in accordance with the Ethical Principles in Animal Research adopted by the Brazilian College of Animal Experimentation (COBEA) and were approved by the Biosciences Institute – Ethics Committee for Animal Research (CEEA). Adult Swiss male mice (30 days old) from a colony at the UNESP Central Animal House facility were used in all of the experiments after a one-week acclimation period in the Animal House of the Department of Pharmacology. Thus, the animals used were approximately 40 to 45 days old. The animals were maintained under controlled temperature (21 ± 2°C) and light (12/12 light/dark cycle) conditions, with food and water *ad libitum* until 2 h prior to the experimental procedures.

### Drugs

Diazepam (DZP, Germed - EMS, Brazil) was used as the standard anxiolytic drug, and imipramine hydrochloride (IM, Sigma-Aldrich, USA) was used as the standard antidepressant drug. Flumazenil (FLU, Flumazil® - Cristália, Brazil) was utilized as a competitive antagonist of benzodiazepine binding, buspirone (BUSP, Sigma-Aldrich, USA) was used as a partial agonist of 5-HT_1A_ receptors, and WAY100635 (WAY, Sigma-Aldrich, USA) was used as a highly selective 5-HT_1A_ antagonist. For the intraperitoneal injections (i.p.), DZP, IM, and BUSP were dissolved in isotonic saline solution (SAL, 0.9%). For oral administration (p.o.), the EO or limonene (d-limonene, 98%, LIM, Sigma-Aldrich, USA), a major component of the EO, were dissolved in 0.01% (v/v) polyoxyethylene sorbitan monooleate (TW - Tween 80®, Sigma-Aldrich) in saline, which was used to treat the control groups. All of the solutions were freshly prepared on the test day and were administered at 10 ml/kg of body weight, except FLU, which was administered at 20 ml/kg due to the concentration (0.1 mg/ml) of its commercial form.

### Behavioral procedures

The behavior of the mice in the light/dark box and forced swim tests was videotaped under white light illumination using a video camera and a computer. The digital video was subsequently reviewed, and behaviors were scored by a highly trained observer who had been blinded to the treatment conditions. In the rotarod test, the behaviors were registered in real time. All of the experimental procedures took place between 9:00 am and 5:00 pm.

### Light/Dark Box Test (LDB)

The apparatus used in our lab has previously been described in detail [28], and our procedure was based on the original protocol proposed by Crawley and Goodwin [29]. Each animal was individually placed in the center of the light compartment facing the dark compartment and observed for 5 minutes after the first entry into the dark compartment. During this period, we recorded the number of shuttle crossings (an activity-exploration index), the number of rearings, and the time spent in the light compartment, which is the most consistent and useful measure for assessing anxiolytic-like activity [30,31].

Mice acutely pretreated (30 min) with EO at 1, 5, 10 or 50 mg/kg and mice repeatedly treated with EO at 1, 5 or 10 mg/kg/day for 14 days were used in this assay, with the experimental session being carried out 30 min after the last treatment. Despite the consistent activity of 1 mg/kg DZP (positive control) after a single treatment, DZP was not effective in modifying the parameters evaluated in the LDB after repeated treatments [28]. For this reason, the positive control group in the repeated treatment procedure received BUSP (10 mg/kg), a partial agonist of 5-HT_1A_ receptors that is recognized as a suitable anxiolytic with a slow onset of action [32].

The LDB test was also used to address a possible contribution from the GABA-benzodiazepine or serotonergic neurotransmission systems in the biological activity of EO. Therefore, to assess the putative contribution of these systems, mice were co-administered EO (5 mg/kg, p.o.) and FLU (2 mg/kg, i.p.) or WAY (0.5 mg/kg, i.p.). FLU, a competitive benzodiazepine antagonist, was administered 15 min after EO (group EO + FLU), while WAY, a specific 5-HT_1A_ receptor antagonist, was administered 15 min before EO (group WAY + EO). The LDB procedure was performed 30 min after the last drug treatment.

### Rotarod Test (RRT)

At the end of the LDB procedure, the mice acutely or repeatedly treated with EO or used to study the EO mechanism of action were immediately placed onto the Rotarod apparatus to verify the integrity of their motor systems. Each animal was placed on a non-slippery plastic rod 3.0 cm in diameter that was rotating at 5 rpm. The mice were classified as “able” or “unable” based on their ability to walk on the rotating bar for 1 min, with a tolerance of up to three falls [33]. To avoid a bias due to an incapacity not related to drug treatment, the mice were assessed in the rotarod test 24 hours before the start of the experimental procedure. Only those animals that performed satisfactorily in this initial assessment were evaluated in the RRT after the LDB.

### Forced Swim Test (FST)

FST is consistently recognized as a model for assessing antidepressant activity [34-36] and our experimental conditions were previously detailed [28]. Briefly, each animal underwent a 15 min pre-test swim in a 13-cm column of water (23 ± 2°C) in a transparent plastic cylinder (20.5 cm diameter). The pre-test session was conducted 24 h prior to the 5-min swim tests, in which the immobility time (in seconds) was determined by a post hoc evaluation of the digital video using the *Etholog 2*.*2* software [37]. Mice treated with oral or inhaled EO were used in the FST.

For oral treatment, the mice received EO at 1, 5, 10 or 50 mg/kg according to the following schedule: the first treatment (T1) was administered 1 h after finishing the pre-test session, with the second and third treatments administered 5 h (T2) and 30 min (T3) before the test session, respectively. The inhaled treatments were given in an acrylic box with cotton swabs soaked in 2 ml of EO at 0.5%, 1.0%, or 2.5%, as described by Almeida et al. [38]. Mice were individually placed into the box for 7 min to receive T1, T2, and T3 following the same time schedule described for oral administration. The positive control group was exposed to the inhalation box with cotton swabs soaked in saline and treated with IM 30 mg/kg (i.p.), while the negative control group was exposed to and treated with saline (i.p.).

### Toxicity and biochemical analyses

The mice treated with 1, 5 or 10 mg/kg EO for 14 days were observed daily for signs and symptoms of toxicity and were periodically weighed to evaluate changes in body weight. Thirty minutes after the last treatment, the mice were submitted to the LDB and the RRT, both of which are described above.

After completion of the behavioral evaluation, blood was collected for biochemical evaluation. The blood samples were collected in tubes without anticoagulant, which had been kept at room temperature for 30 min, and were centrifuged at 4,000 rpm for 20 min at 4°C. The serum samples were aspirated off and stored at −80°C until analysis in a Cobas Mira Plus® Chemistry Analyzer (Roche Diagnostic Systems, USA). The biochemistry parameters measured were aspartate aminotransferase, alkaline phosphatase, urea, albumin, creatinine, total protein, cholesterol, and triglycerides.

### Neurochemical evaluation

After 30 min of acute treatment with TW or EO (5 mg/kg), the mice were euthanized and the brains rapidly dissected (not more than 3 min after extraction) on ice. The striatum, hypothalamus, pons, and frontal cortices were isolated, weighed, and stored in liquid nitrogen. For the neurochemical analyses, the tissues were homogenized in 0.1 M perchloric acid by manual sonication, centrifuged at 10,000 rpm for 20 minutes to remove the supernatant, and stored at - 80°C until the determination of monoamine levels.

The levels of neurotransmitters and their metabolites (dopamine (DA), 3,4-dihydroxyphenylacetic acid (DOPAC) and homovanillic acid (HVA), and serotonin (5HT) and 5-hydroxyindoleacetic acid (5HIAA)) were measured by reversed-phase high performance liquid chromatography (HPLC) using a system (model 6A, Shimadzu, Kyoto, Japan) with an electrochemical detector as described elsewhere [39]. Briefly, 20-μl samples were loaded into a sample injector, and the mobile phase was delivered at a constant rate of 1.2 ml/min. The runtime for each sample was 15 min, and the concentrations of all neurotransmitters and their metabolites were expressed as nanograms per gram of tissue (ng/g).

### Statistical analysis

The quantitative data from the behavioral procedures were subjected to Kruskal-Wallis non-parametric variance analysis followed by the Mann-Whitney test when appropriate. The proportions in the RRT were compared by Fisher’s exact test. The biochemical data were analyzed by a one-way ANOVA followed by Dunnett’s Multiple Comparison test, while the neurochemical data were analyzed with an unpaired t-test. All of the statistical analyses was made using GraphPad InStat® version 3.02. The treated and TW groups were compared, and differences were considered significant when p ≤ 0.05.

## Results

### EO composition

Table 1 shows the results from the gas chromatography coupled with mass spectrometry analysis of the *C*. *aurantium* EO (yield: 0.50% v/w). The main EO compound was identified by retention time and the Kovats retention index [40] as monoterpene limonene (98.66%). The other compounds detected in the EO included β-pinene (0.41%) and β-myrcene (0.53%).

**Table 1:** **Chemical composition of the *C*. *aurantium *L. EO obtained by gas chromatography coupled with mass spectrometry**

**Constituent**	**RT**	**RI**	**%**
β-pinene	6.869	977	0.41
β -myrcene	7.261	991	0.53
Limonene	8.539	1037	98.66
ni	11.072	1100	0.41

RT, retention time; RI, retention index (experimental); ni, not identified.

### LDB procedure

The LDB was used to access the anxiolytic-like effect of the EO after acute and repeated treatment, as well as to investigate the effect of interference on the GABAergic and serotonergic neurotransmission systems on the anti-anxiety activity of the EO.

**Figure 1:**
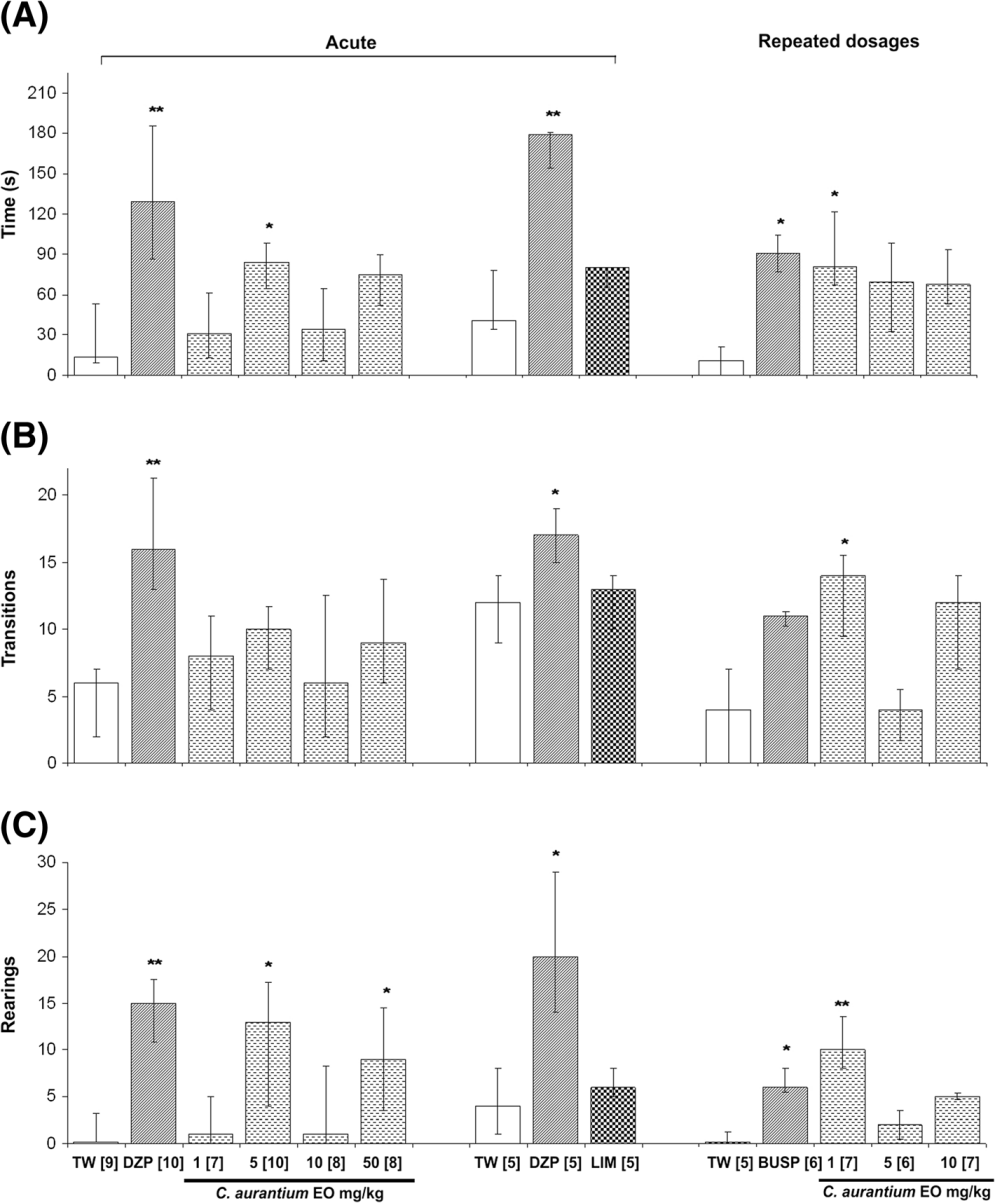
**The effect of acute or repeated treatments on (A) the time (s) spent in the illuminated chamber, (B) the number of transitions between chambers and (C) the number of rearings evaluated in the light/dark box test.** The data are presented as the median and interquartile range (Q1-Q3). TW: vehicle, 10 ml/kg (p.o.); DZP: diazepam, 1 mg/kg (i.p.); EO: *Citrus aurantium* essential oil, 1, 5, 10 or 50 mg/kg (p.o.); LIM: d-limonene, 5 mg/kg (p.o.); BUSP: buspirone, 10 mg/kg (i.p.). The numbers in brackets indicate the number of mice in each group. *p ≤ 0.05, **p ≤ 0.01 in relation to the TW group (Kruskal-Wallis followed by Mann-Whitney U-Test).

The results of the LDB (Figure 1, left panel) revealed a significant increase in the amount of time spent in the light chamber after the administration of a single dose of 5 mg/kg EO when compared to the TW group. An increase in exploratory parameters (the number of rearings) was observed after treatment with either DZP or EO (5 or 50 mg/kg). The major compound in the EO (limonene, 5 mg/kg, p.o.) was also evaluated after acute treatment and did not modify behavioral parameters in the LDB (Figure 1, central panel). Repeated treatment with 1 mg/kg EO for 14 days modified the amount of time spent in the illuminated side and the number of transitions and rearings (Figure 1, right panel).

**Figure 2:**
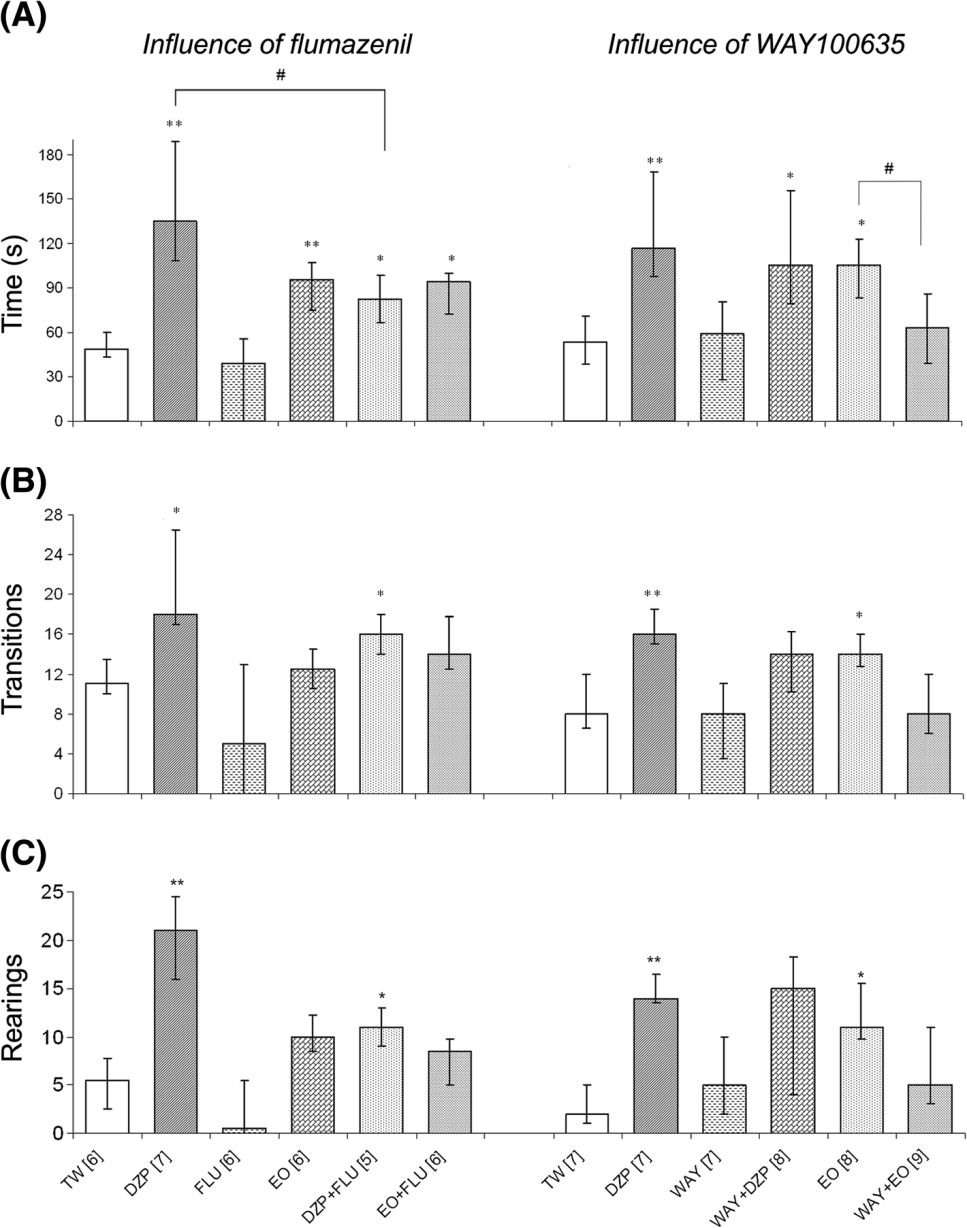
**The effect of blocking the benzodiazepine-GABA (left) or 5-HT**_**1A**_**(right) receptors on (A) the time (s) spent in the illuminated chamber, (B) the number of transitions between chambers and (C) the number of rearings evaluated in the light/dark box test.** The data are presented as the median and interquartile range (Q1-Q3). TW: vehicle, 10 ml/kg (p.o.); DZP: diazepam, 1 mg/kg (i.p.); EO: *C*. *aurantium* essential oil, 5 mg/kg (p.o.); FLU: flumazenil, 2.0 mg/kg (i.p.); WAY: WAY100635, 0.5 mg/kg (i.p.). The numbers in brackets indicate the number of mice in each group. *p ≤ 0.05, **p ≤ 0.01 in relation to the TW group; # p ≤ 0.05 between compared groups (Kruskal-Wallis followed by Mann-Whitney U-Test).

The results of the GABAergic and serotonergic interference study are presented in Figure 2. As expected, FLU, a competitive antagonist of the benzodiazepine receptor, partially reversed the DZP effect, as the co-administration of these compounds (DZP + FLU) significantly decreased the magnitude of the DZP effect. Conversely, the EO effect was not affected by the co-administration of FLU (OE + FLU), suggesting that the EO does not function through the GABA-benzodiazepine receptor complex.

A similar approach was used to evaluate interference with the 5HT_1A_ serotonin receptor. Administering WAY with the EO (WAY + EO) reversed all of the effects of the EO in the LDB. Thus, it appears that the serotonergic system, through 5HT_1A_ receptor, is linked to the anxiolytic-like action of the EO. To rule out the possibility of a nonspecific effect, WAY was also co-administered with DZP (WAY + DZP) without any observable effect.

### RRT procedure

The results presented in Table 2 include the number of “unable” mice in relation to the total number of mice evaluated in each experimental group. Acute or repeated treatment with the EO had no effect on the ability of the mice to remain on the rotating bar. In the same way, the individual or combined administration of the benzodiazepine or 5HT_1A_ receptor agonists and antagonists and the EO did not interfere with motor coordination as evaluated by the RRT.

**Table 2:** Effects of distinct treatments on motor coordination as evaluated by the rotarod test

**Acute treatment**				**Repeated treatment**	
**Group**	**Fraction**	**Group**	**Fraction**	**Group**	**Fraction**
TW 0.01%	1/7	FLU 2	0/5	TW 0.01%	0/5
DZP 1	0/8	WAY 0.5	1/5	BUSP 10	0/6
EO 1	0/5	DZP 1 + FLU 2	0/4	EO 1	0/5
EO 5	0/8	WAY 0.5 + DZP 1	1/5	EO 5	0/5
EO 10	1/6	EO 5 + FLU 2	0/4	EO 10	0/7
EO 50	0/6	WAY 0.5 + EO 5	0/5		

The data are presented as the fraction of the total number of animals in each experimental group that were unable to perform the test after acute or repeated (14 days) treatments. TW: vehicle; DZP: diazepam; OE: *C*. *aurantium* essential oil; BUSP: buspirone; FLU: flumazenil; WAY: WAY100635. The doses are presented in mg/kg. The differences were compared in relation to the TW group using Fisher’s exact test.

### FST procedure

The results of the forced swim test after the oral or inhaled administration of the EO are presented in Table 3. The different doses or routes of EO administration showed no signs of interference with immobility time, which is the main parameter indicating antidepressant activity in this experimental procedure.

**Table 3:** **Effects of oral or inhaled *C*. *aurantium *EO on immobility time (s) in the forced swim test**

**Oral route**			**Inhalatory route**		
**Treatment**	**n**	**Immobility (s)**	**Treatment**	**n**	**Immobility (s)**
TW	5	226 (225–231)	SAL	5	207 (203–219)
IM	6	156 (120–165) *	IM	6	167 (164–171) *
EO 1 mg/kg	6	222 (215–238)	EO 0.5%	8	215 (207–221)
EO 5 mg/kg	7	234 (217–241)	EO 1.0%	8	194 (179–208)
EO 10 mg/kg	8	238(224–255)	EO 2.5%	8	220 (175–234)
EO 50 mg/kg	6	217 (206–222)			

The data are expressed as the median and interquartile range (Q1-Q3). TW: vehicle; SAL: isotonic saline solution; IM: imipramine hydrochloride, 30 mg/kg (i.p.); EO: *C*. *aurantium* essential oil. * p ≤ 0.05 in relation to the corresponding TW or SAL group (Kruskal-Wallis followed by Mann-Whitney U-Test).

### Toxicity and biochemical analyses

The 14-day EO treatment did not induce any observable signs of toxicity; changes in body weight; or abnormalities in the serum levels of aspartate aminotransferase, alkaline phosphatase, urea, albumin, creatinine, total protein, or triglycerides. The only parameter that was altered was the serum total cholesterol, which was significantly reduced by the 10 mg/kg EO treatment (Table 4).

**Table 4:** **Changes in body weight and biochemical parameters after 14 days of treatment with *C*. *aurantium *EO**

**Treatment**	**n**	**Body weight (g)**		**Biochemical parameters**							
		**Day 1**	**Day 14**	**UR (mg/dL)**	**CR (mg/dL)**	**TPRO (g/dL)**	**ALB (g/dL)**	**AST (UI/L)**	**AP (UI/L)**	**CHOL (mg/dL)**	**TRGL (mg/dL)**
Tween	6	35 ± 4	43 ± 7	64.5 ± 10.1	0.3 ± 0.1	7.0 ± 0.1	3.9 ± 0.3	320.4 ± 85.5	164.8 ± 74.2	158.8 ± 35.2	173.8 ± 42.5
EO 1 mg/kg	7	34 ± 6	41 ± 10	64.4 ± 13.7	0.3 ± 0.2	7.9 ± 1.9	4.5 ± 0.9	334.2 ±5 2.7	131.4 ± 44.7	131.4 ± 27.2	143.6 ± 50.8
EO 5 mg/kg	6	34 ± 2	42 ± 4	65.3 ± 13.7	0.2 ± 0.1	6.9 ± 0.8	4.2 ± 0.9	377.4 ± 83.6	161.9 ± 64.0	135.2 ± 15.6	130.8 ± 37.5
EO 10 mg/kg	7	34 ± 4	40 ± 5	64.1 ± 11.9	0.2 ± 0.0	6.9 ± 0.6	3.9 ± 0.4	309.5 ± 45.2	123.3 ± 34.9	121.9 ± 20.4 *	132.7 ± 33.4

The data are presented as the mean ± SD. UR (urea), CR (creatinine), TPRO (total protein), ALB (albumin), AST (aspartate aminotransferase), AP (alkaline phosphatase), CHOL (cholesterol), TRGL (triglycerides). * p ≤ 0.05 in relation to the control group (one-way ANOVA followed by the Dunnett’s Multiple Comparisons Test).

### Neurochemical evaluation

Acute treatment with *C*. *aurantium* EO did not change the levels of neurotransmitters or their metabolites (DA, DOPAC, HVA, 5HT, and 5HIAA) in the cortex, the striatum, the pons or the hypothalamus (Table 5).

**Table 5:** **Neurotransmitter levels and their metabolites (ng/g) after acute oral treatment with *C*. *aurantium *EO (5 mg/kg)**

**Structures and treatments**								
	**Cortex**		**Striatum**		**Pons**		**Hypothalamus**	
	**TW**	**EO**	**TW**	**EO**	**TW**	**EO**	**TW**	**EO**
DA	845.6 ± 239.9 (10)	895.6 ± 321.3 (7)	8324.3 ± 517.1 (11)	8331.2 ± 1040.1 (7)	15.2 ± 3.8 (10)	18.6 ± 6.2 (7)	53.1 ± 3.8 (10)	58.3 ± 5.8 (6)
DOPAC	94.6 ± 15.9 (12)	85.6 ± 17.4 (8)	437.4 ± 44.5 (11)	415.5 ± 49.9 (7)	11.6 ± 1.6 (11)	8.4 ± 1.1 (8)	58.6 ± 5.6 (12)	64.2 ± 4.7 (8)
HVA	427.3 ± 80.4 (12)	567.8 ± 112.5 (8)	1121.9 ± 96.8 (11)	1384.9 ± 193.5 (7)	317.2 ± 87.9 (11)	388.3 ± 141.3 (8)	236.2 ± 36.8 (12)	388.8 ± 74.5 (8)
5HT	1246.9 ± 85.8 (12)	1270.1 ± 81.0 (8)	1686.3 ± 102.4 (11)	1665.9 ± 170.9 (7)	2782.4 ± 259.8 (11)	2075.4 ± 309.9 (8)	1660.1 ± 126.2 (12)	1599.2 ± 113.9 (8)
5HIAA	192.7 ± 17.5 (12)	229.3 ± 33.7 (8)	317.1 ± 30.9 (11)	320.3 ± 40.0 (7)	879.2 ± 69.5 (10)	644.1 ± 76.4 (7)	341.7 ± 20.9 (12)	310.9 ± 19.4 (8)

TW (tween). The data are presented as the mean ± SEM. Any differences were compared with an unpaired t-test. The number of animals is different for each group because some structures could not be evaluated due to technical reasons.

## Discussion

Traditional populations usually consider the leaves and flowers of *Citrus* species as a useful decoction [12,14] or infusion [13,15] to treat nervous system disturbances. Previously our group demonstrated the sedative and anxiolytic-like effects of *C*. *aurantium*, *C*. *latifolia*, and *C*. *reticulata* EO in mice [23-25].

Therefore, considering that the rate of comorbidity between anxiety and depression is approximately 60 to 70% in the general population [41] and that studies indicate that these conditions have common genetic origins [42], it is reasonable to propose that the same substance can act as a therapeutic for both disorders. The present work not only investigated the mechanism of action underlying the anxiolytic-like effect of *C*. *aurantium* EO but also investigated the antidepressant-like activity of this EO.

The major compound present in the EO was limonene, followed by β-myrcene. Both of these compounds are biologically active in the central nervous system, as mice treated with these compounds have shown decreases in spontaneous activity, rearing, and grooming in an open field test, and the compounds increased barbiturate sleeping time [43]. In the present work, limonene did not modify the parameters evaluated in the LDB at a dose of 5 mg/kg, which corresponds to the level present in the effective dose of the EO (5 mg/kg). These results corroborate previous reports [43] in which limonene was not able to modify the parameters of anxiolytic-like activity evaluated in the elevated plus maze procedure. These findings strengthen the idea that, in herbal preparations, the interactions between compounds often result in biological activity that is greater than the activity of isolated compounds, as noted earlier [44].

The 5HT system has been implicated in the modulation of anxiety levels, and some components of this system promote anxiety while others reduce its symptoms [45]. Since the introduction of buspirone as a novel therapeutic agent for the treatment of anxiety, considerable interest in its therapeutic role in anxiety and mood disorders has been generated [2]. Buspirone is a partial agonist of 5-HT_1A_ autoreceptors that acts as an antagonist at certain postsynaptic 5-HT_1A_ receptor sites. While reports on the effect of the acute administration of buspirone are inconclusive, chronic treatment causes an anxiolytic-like effect [4,5].

According to our results, the EO showed anxiolytic effects after acute (5 mg/kg) or 14-day repeated (1 mg/kg/day) treatments, increasing all parameters evaluated in the LDB. As previously reported for EO [23], the profile of dose-response curve is not monotonic as usually seen in classical pharmacology. In a monotonic curve, the sign (negative or positive) of the slope is maintained throughout the entire dose range. Conversely, in nonmonotonic dose-response curve the slope changes sign at some point along the range of doses, resulting in a U- or inverted U-shape. In more complexes cases, a nonmonotonic curve assumes a multiphasic shape, in which the slope changes the sign in multiple points along the curve. This complex situation emerges from our results given that the dose of 5 mg/kg shows a significant effect similar in magnitude to the dose of 50 mg/kg, with no effect at the lower (1mg/kg) and intermediate (10 mg/kg) doses.

This phenomenon has puzzled researchers for more than 50 years and recently a comprehensive overview discusses and contributes to its better understanding [46]. In spite of the main focus under endocrinology field, examples have also emerging in different areas of research such as toxicology, epidemiology and pharmacology [46,47]. Among the several putative mechanisms which produce nonmonotonic responses (in cells, tissues, and animals) stand out the cytotoxicity, cell-specific and tissue-specific receptors and cofactors, receptor competition, superimposition of monotonic dose responses and other events related with responses of a biological system caused by products that have a complex mixture of substances [46,48], including essential oils.

The anxiolytic-like effects of the acute treatments were not antagonized by FLU, a benzodiazepine antagonist, but these effects were antagonized by the 5-HT_1A_ specific antagonist WAY. Considering that benzodiazepines are not effective in the LDB after chronic treatment [28] and the reduction in the anxiolytic-like effect of the EO after WAY administration, we can infer that the EO functions through interactions with the serotonergic system. However, we cannot discard the possibility that the EO or its metabolites interact with other neurotransmission systems. Increased 5HT and DA synthesis were observed in the prefrontal cortex and hippocampus of mice after the inhalation of lemon oil vapor (a species from the *Citrus* genus), suggesting that lemon oil vapor possibly affects the response to DAnergic activity by modulating the 5HTnergic system [49]. In our assessment of DA, 5HT, and their metabolites, no alterations in the levels of these neurotransmitters were observed in the cerebral areas evaluated after acute treatment with the EO. Higher EO doses and/or repeated treatment could reveal this effect on neurotransmitter systems.

Contradicting our expectations and previous reports [20,21], the EO was unable to modify immobility time in the FST, an experimental model of depression. The use of 5-HT_1A_ ligands for the treatment of depressive disorders shows inconsistent results [50]. This inconsistency may be related to differences in the intrinsic activity and potency of 5-HT_1A_ drugs that appear to depend on the brain region [4].

Despite the effect observed in a study treating rats with the same concentration used in our procedure [16], we could not find an anxiolytic-like effect after inhaled exposure to EO. Similarly, no antidepressant-like effect was found in the FST after the inhalation of EO.

Finally, in agreement with a subchronic 28-day toxicity study of *C*. *aurantium* extracts in mice [51], the 14-day repeated EO treatment in this study showed no deleterious consequences. Daily inspection and periodic weighing showed no differences in general aspect, body weight, or the biochemical parameters of the experimental groups, except for a decrease in total cholesterol. This finding is not unexpected because polymethoxylated flavones, which are compounds found in a variety of citrus fruits, have been shown to cause a hypolipidemic response in hamsters with experimental hypercholesterolemia [52], and the supplementation of 2% d-limonene, a major compound in *C*. *aurantium*, to a high fat diet in Wistar rats reversed the diet-induced changes in lipid levels and lipid peroxidation [53].

## Conclusion

*C*. *aurantium* EO possesses a significant anxiolytic-like activity, and the present results strongly suggest the involvement of 5-HT_1A_-receptors. We believe that these results are promising, as EO treatment might be considered a complementary therapy for the treatment of anxiety disorders. However, further studies are necessary to explore the detailed mechanism of EO action on binding sites. Moreover, the EO appears to be well tolerated, as none of the different doses caused alterations or showed signs of toxicity.

## Competing interests

All authors declare that they have no competing interests.

## Authors’ contributions

CARAC participated in the design of the study, carried out all the experimental procedures and drafted the manuscript. TCC and BOC participated in experimental protocols and analyzed the behavioral data. RKT made and supervised all biochemical analyzes. JCF participated in the design of the study and made the neurochemical analyses. MC supervised the entire project, participated in its design and statistical analysis and helped to the final form of the manuscript. The work was part of the Ph.D. dissertation of CARAC and the graduation work of TCC and BOC. All of the authors significantly contributed to and have approved the final manuscript.

## Pre-publication history

The pre-publication history for this paper can be accessed here:

http://www.biomedcentral.com/1472-6882/13/42/prepub
